# Environmental controls on the distribution and diversity of lentic Chironomidae (Insecta: Diptera) across an altitudinal gradient in tropical South America

**DOI:** 10.1002/ece3.1833

**Published:** 2015-12-10

**Authors:** Frazer Matthews‐Bird, William D. Gosling, Angela L. Coe, Mark Bush, Francis E. Mayle, Yarrow Axford, Stephen J. Brooks

**Affiliations:** ^1^ Environment, Earth & EcosystemsThe Open UniversityWalton HallMilton KeynesMK76AAUK; ^2^ Biological SciencesFlorida Institute of Technology150 West University BoulevardMelbourneFlorida32901; ^3^ Palaeoecology & Landscape EcologyInstitute of Biodiversity & Ecosystem Dynamics (IBED)University of AmsterdamScience Park 9041098 XHAmsterdamThe Netherlands; ^4^ Department of Geography and Environmental Science (SAGES)Centre for Past Climate ChangeUniversity of ReadingReadingRG6 6ABUK; ^5^ Department of Earth and Planetary SciencesNorthwestern University2145, Sheridan RoadEvanstonIllinois60201; ^6^ Department of Life SciencesNatural History MuseumCromwell RoadLondonSW7 5BDUK

**Keywords:** Andes, chironomids, climate change, diversity, lakes

## Abstract

To predict the response of aquatic ecosystems to future global climate change, data on the ecology and distribution of keystone groups in freshwater ecosystems are needed. In contrast to mid‐ and high‐latitude zones, such data are scarce across tropical South America (Neotropics). We present the distribution and diversity of chironomid species using surface sediments of 59 lakes from the Andes to the Amazon (0.1–17°S and 64–78°W) within the Neotropics. We assess the spatial variation in community assemblages and identify the key variables influencing the distributional patterns. The relationships between environmental variables (pH, conductivity, depth, and sediment organic content), climatic data, and chironomid assemblages were assessed using multivariate statistics (detrended correspondence analysis and canonical correspondence analysis). Climatic parameters (temperature and precipitation) were most significant in describing the variance in chironomid assemblages. Temperature and precipitation are both predicted to change under future climate change scenarios in the tropical Andes. Our findings suggest taxa of Orthocladiinae, which show a preference to cold high‐elevation oligotrophic lakes, will likely see range contraction under future anthropogenic‐induced climate change. Taxa abundant in areas of high precipitation, such as *Micropsectra* and *Phaenopsectra*, will likely become restricted to the inner tropical Andes, as the outer tropical Andes become drier. The sensitivity of chironomids to climate parameters makes them important bio‐indicators of regional climate change in the Neotropics. Furthermore, the distribution of chironomid taxa presented here is a vital first step toward providing urgently needed autecological data for interpreting fossil chironomid records of past ecological and climate change from the tropical Andes.

## Introduction

Chironomids are one of the most widely distributed free‐living holometabolous insects in the world, with an estimated 8000–20,000 species globally (Armitage et al. 1995). Chironomids are a keystone group (Jones and Grey 2004; Ólafsson and Paterson 2004) and a vital nexus between primary producers and secondary consumers, playing a key role in the cycling of nutrients through the freshwater ecosystem (Porinchu and Macdonald 2003). Changes in abundance or distribution to such an integral component of the aquatic food web will have cascading effects through an ecosystem (Petchey et al. 1999). Consequently, chironomids have long been used as bioindicators in lakes and rivers (Thienemann 1922; Walker et al. 1991; Milošević et al. 2013). On long timescales (100–1000 years), analyses of subfossil chironomid larval remains have shown that chironomid taxa rapidly track changing environmental conditions (Brooks and Birks 2001). Across Eurasia (Brooks and Langdon 2014; Engels et al. 2014; Heiri et al. 2014) and North America (Walker et al. 1991; Fortin et al. 2015), chironomids have been used as quantitative palaeoclimate proxies and have provided important contributions to our understanding of Late Glacial/Holocene (last *c*. 21 kyrs) environmental and climate change in these regions. In the tropics, however, both chironomid‐based biomonitoring and quantitative palaeoecology have lagged behind. To a large extent, this can be attributed to a paucity of taxonomic knowledge of larval chironomids from these regions, inadequate autecological information that could attribute important bioindicator information to sensitive taxa, and the logistical difficulties in sampling and surveying a large number of lakes in these regions in order to acquire this information (Verschuren and Eggermont 2006).

Analysis of larval head capsules, derived from the shed skins of developing larvae, found in surface sediments provides an efficient way of sampling multiple sites while obtaining representative samples of the chironomid fauna (Heggen et al. 2012). Studies of chironomid larval distributions from surface sediment samples across multiple lakes in the tropics are limited to a few studies from tropical East Africa (Eggermont and Verschuren 2007), Central America (Pérez et al. 2013; Wu et al. 2014), and Australia (Dimitriadis and Cranston 2001; Chang et al. 2015). Broad distributional data from chironomid larval remains in South America are available from the high latitudes where lentic chironomid larval remains were recovered from surface sediment samples in Patagonian lakes (e.g., Massaferro and Larocque 2013; Massaferro et al. 2014). However, no previous studies have assessed the broad larval distributions of lentic chironomid species from the inner (*c*. 0–11°S) and outer (*c*. 11–23°S) tropical Andes, or the immediate lowlands (Amazonia). To date, across the tropical Andes, research has focused on lotic chironomid species (Roque et al. 2010a; Prat et al. 2011; Loayza‐Muro et al. 2014), and currently, there is little autecological data available for the interpretation of lentic chironomid palaeoecological records in the region (e.g., Williams et al., 2012).

Climate change (over the last *c*. 150 years) has had environmental consequences at physiological, biogeographical, and phenological levels for species around the world (Hughes 2000). Species distributions along altitudinal and latitudinal gradients are particularly influenced by temperature change (Walther et al. 2002). To predict possible responses to changing climate under various future climate scenarios, it is important to understand species realized and potential climatic envelopes (Notaro et al. 2012). Aquatic insects, such as chironomids, have life cycles strongly dependent upon temperature and the distribution of species is expected to alter with increased warming (Sweeney et al. 1991 1992). Furthermore, mountain ecosystems are some of the most sensitive environments to climate change, as many organisms at their climatic limits inhabit narrow ecological niches (Gottfried et al. 2012). The tropical Andes is one of the world's most important biodiversity hotspots (Myers et al. 2000) and is vulnerable to future global change (Bellard et al. 2014). Close monitoring is needed to evaluate the biodiversity response to future change and provide empirical observations from which to test projections (Bellard et al. 2014). A lack of data on chironomid ecology and distribution limits our ability to observe and understand future environmental and climate change within aquatic ecosystems across this important region.

In order to address the lack of faunal studies in this region, we (1) identify the diversity and abundance of chironomid from 0°S to 17°S in 59 lakes, (2) determine the primary variables (physical environment, lake chemistry, and climate) that influence chironomid distribution, and (3) identify important indicators for monitoring future environmental change. These data will also provide autecological data for the interpretation of chironomid paleoecological records in the tropical Andes.

## Materials and Methods

### The distribution of the studied lakes

**Figure 1:**
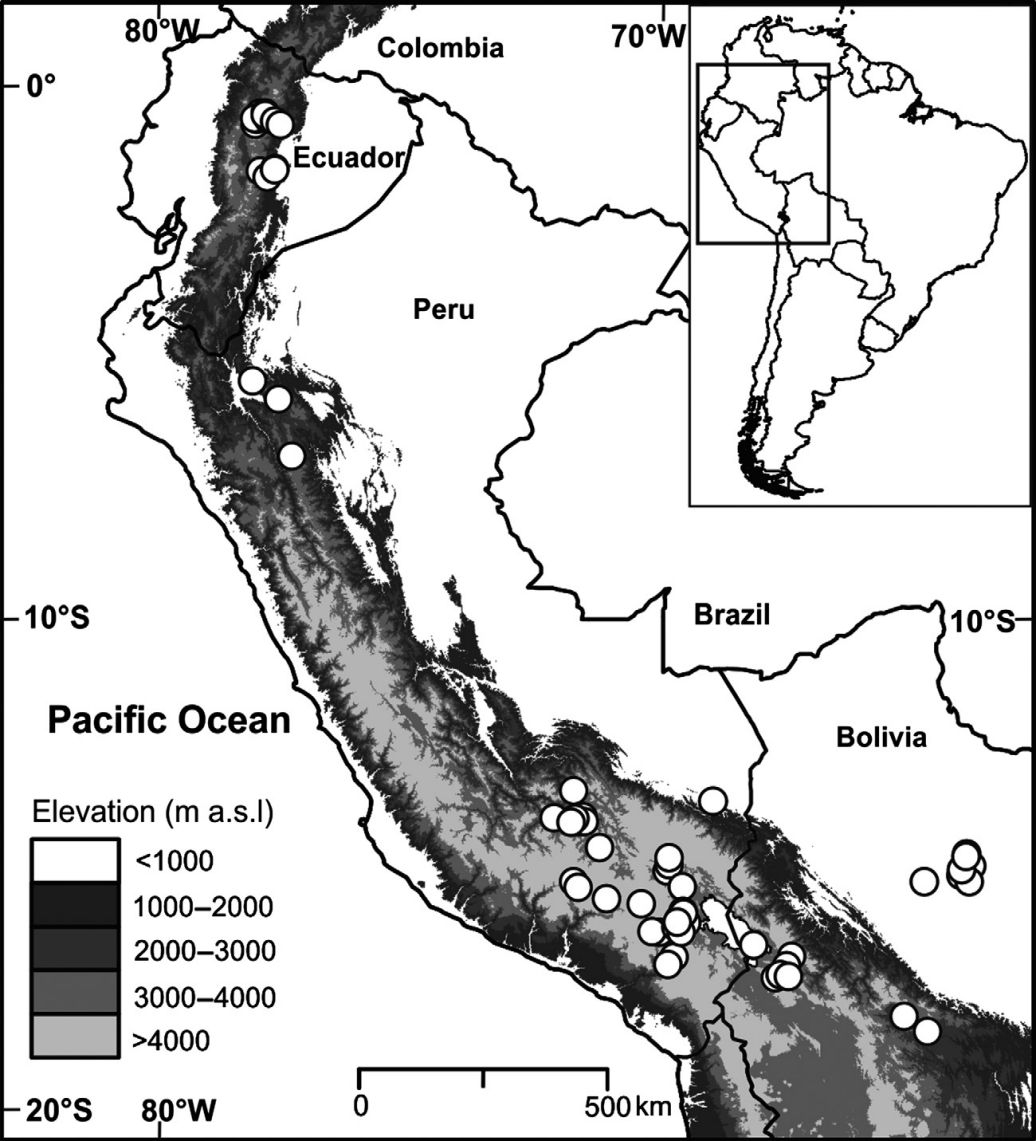
Map of South America showing the extent of the study sites. Studied lakes are in the eastern inner and outer Neotropics between 0 and 17°S and 150 and 4655 m a.s.l. White circles denote study lakes from the Andes to lowland Amazonia.

Lake surface sediment samples for chironomid analysis and associated environmental data were obtained from 59 lakes (15 from Bolivia, 32 from Peru, and 12 from Ecuador) between 2004 and 2013 (Fig. 1). The study region covers an altitudinal gradient of 4505 m, from 150 (m a.s.l.) to 4655 (m a.s.l.), between 0.1–17°S and 64–78°W (Fig. 1, Table 1). No lakes were sampled between 1000 and 2000 m a.s.l. Seven of the sites are in lowland Bolivian Amazonia (≤200 m a.s.l.), in tropical/subtropical grass savanna–shrubland (Olson et al. 2001) (Biome 1) where mean annual temperature (MAT) ranges between *c*. 24 and 25°C and mean annual precipitation (MAP) varies between *c*. 1760 and 1970 mm/year (Hijmans et al. 2005). Nine lakes lie at mid‐elevations (1000–3000 m a.s.l.), within the tropical/subtropical moist broadleaf forest (Olson et al. 2001) of Ecuador and Peru (Biome 2) where MAT ranges from *c*. 13 to 21°C and MAP ranges from *c*. 1110 to 4420 mm/year (Hijmans et al. 2005). Only one lake in the dataset, Tendamina, was located within the tropical/subtropical dry broadleaf forest of Peru (Biome 3, MAT = *c*. 22°C, MAP = *c*. 1206 mm/year). The remaining 42 lakes are high Andean (≥3000 m.a.s.l.) spread across Peru, Ecuador, and Bolivia. Due to the effects of extreme elevations (>4500 m a.s.l.), and the large latitudinal gradient (*c*. 17°), a significant range of climatic conditions exists between the studied high‐elevation lakes. At these elevations, the biome is montane grass and shrubland (Olson et al. 2001) (Biome 4), MAT ranges from *c*. −1 to 17°C (Hijmans et al. 2005). MAP varies between 470 mm/year in the southern section of the transect and 1430 mm/year in northern regions (Hijmans et al. 2005). All the studied lakes were relatively shallow; mean water depth of all the study sites was 4.9 m, the deepest lake was 14.4 m, and the shallowest is 0.25 m.

**Table 1:** Environmental variables and chironomid richness (rarefied to 23 specimens). Latitude and longitude are noted in decimal degrees. Water temperature (WT) was recorded at point of sample collection. Mean annual temperature (MAT), mean January temperature (MJT), and mean annual rainfall (MAP) are based on 30‐ to 50‐year averages of satellite and meteorological station observations (Hijmans et al. 2005)

Lake	Elevation (m a.s.l)	Latitude (S)	Longitude (W)	Depth (m)	pH	Conductivity (*μ*s)	LOI (%)	WT (°C)	MAT (°C)	MJT (°C)	MAP (mm)	Hills N2	Taxon Richness	N° Head Capsules	Biome
Estrellani	4655	16.4103	68.1878	5.2	7.3	41.8	5.4	6.4	3.5	4.7	625	4.4	4.8	60	Biome 4
PLS‐4‐A	4612	14.9897	72.3342	7.1	7.6	13.7	22.9	10.4	0.8	2.3	728	4.2	4.6	77	Biome 4
PLS‐3‐A	4594	15.1025	72.2544	6.6	7.6	19.2	26.2	9.5	3.3	4.9	820	7.1	7.4	85	Biome 4
PLS‐6‐A	4527	14.7864	70.5578	8.6	8	47.5	23.7	10.1	7.2	9.5	683	3.9	4.4	112	Biome 4
Asiruni	4521	16.4189	70.4308	0.5	9.6	189.5	7.2	10.2	3.2	5	603	1.5	1.7	98	Biome 4
PLS‐8‐A	4486	14.6653	70.5242	10.9	7.8	48.8	51.9	10	6.2	8.5	685	2.6	3.1	44	Biome 4
Jacunarini	4425	16.5661	70.545	1	10.3	437.3	45	8.4	3.6	5.4	599	1.4	1.5	129	Biome 4
Calzada	4415	15.9447	70.6994	12.4	8.7	340.4	12.5	12.1	3.8	5.6	714	5.4	5.3	138	Biome 4
PLS‐10‐A	4280	15.3125	71.7161	12.3	8.9	142.1	24.7	11.8	5.1	6.9	774	7.8	7.4	86	Biome 4
M.Kkota	4269	16.5914	68.2875	5.4	8.6	161.6	8.9	5.7	6.1	6.4	609	4.2	4.4	93	Biome 4
L.Sara Cocher	4183	15.9381	70.8464	2.2	8.3	1038.7	11.5	14.7	5	6.9	718	6.4	5.9	96	Biome 4
Mogotes	4094	0.3772	78.2964	2.2	7.2	200	19.4	9.9	4.3	4.5	1345	2.7	3.6	36	Biome 4
PLS‐13‐A	4083	13.7561	72.1589	6.6	7.8	32.8	80.4	12.1	7.3	8.3	769	5.1	6.1	23	Biome 4
PLS‐7‐A	4081	14.5258	70.52	5.1	7.9	20.5	33.6	8.4	5.7	7.9	694	7	7.7	70	Biome 4
Sucus	4068	0.5917	78.3889	25	5.8	182	17.9	7.6	5.1	4.4	1300	1.8	2.2	68	Biome 4
Quori	4056	13.7747	72.705	18	7.6	36.3	ND	10.8	7.4	8.5	736	5.9	6.4	34	Biome 4
M.pungo big	3979	0.5003	78.3542	10	8.6	193	6.51	11.7	5.2	5.5	1201	2.5	2.7	35	Biome 4
M.pungo small	3957	0.4861	78.4	0.5	9.5	250.7	42.2	15	5.2	5.5	1201	1.5	1.9	69	Biome 4
Pocuna	3914	15.7083	70.3408	3	8.2	82.9	12.4	14.4	9.2	10.3	613	4.6	4.6	101	Biome 4
Guambicocha	3898	0.3783	78.1986	14.4	6.7	203	10.6	10.9	5.8	5.8	1402	6.6	8.2	36	Biome 4
PLS‐9‐A	3895	15.4081	71.0544	1.3	9.4	3205	31.5	14.7	7.8	10	687	3.8	3.6	73	Biome 4
Patos	3887	0.4978	78.1392	1.3	6.5	170	6.8	15.5	5.7	5.9	1429	2.2	2.7	55	Biome 4
Khotana	3885	16.8111	68.5044	1	9.9	264.9	8.9	11.8	7.8	10.2	607	1.7	1.9	37	Biome 4
Jaluncocha	3855	15.855	70.3867	1.3	7.7	881	13.9	13.5	9	10.3	612	3.7	4	125	Biome 4
Salinas	3854	15.0875	70.2736	1.3	10	51.8	8.4	13.9	8.5	10.1	677	5.3	5.5	64	Biome 4
Umayo	3853	15.7656	70.1897	3.2	9.6	1103	27	13.1	8.7	10.1	616	5.1	5	152	Biome 4
Chacas	3851	15.5564	70.2628	4	9.2	218.7	19.8	18.6	9.3	10.7	635	4.6	4.8	144	Biome 4
Umpata	3850	15.5864	70.2403	2	9.9	485	30.3	16.4	8.9	10.4	636	2.7	3.3	104	Biome 4
Aquihui	3846	16.1842	68.9111	2.4	8.2	310.3	23.7	11	8.5	9.8	680	2.8	3.1	98	Biome 4
L.Sollata	3845	15.7608	70.3653	0.25	9.8	1196	8.6	9	8.8	10.2	613	6.5	6.9	104	Biome 4
Lake 3	3845	15.9519	70.2881	0.25	9.1	5.97	12.8	23.8	8.8	10.3	614	5.5	5.7	68	Biome 4
Banos	3821	0.4078	78.1986	0.5	7.5	194.3	13.3	7.1	5.7	6.1	1347	4.2	5.5	41	Biome 4
Lake 27	3796	16.7494	68.4025	1.2	8.1	189	9.2	12.2	7.7	10	566	3.9	4.2	109	Biome 4
PLS‐12‐A	3790	14.3422	71.85	10.9	8.2	1002	26.5	14.6	4.8	5.5	834	1.8	2.3	88	Biome 4
Lake 22	3750	16.7881	68.2531	7.2	7.4	329.3	21.2	11.9	9.2	11	544	4.4	4.7	90	Biome 4
PLS‐1‐A	3728	13.8506	72.2272	10.2	7.8	255.4	6.6	17.3	7.7	8.1	775	4.1	4.1	116	Biome 4
Piuray	3703	13.8442	72.1269	13	9.7	336.8	6.2	13.7	9.4	10.4	710	3.6	4.5	56	Biome 4
Larrati	3586	17.5442	66.0503	ND	ND	ND	7.6	ND	9.3	10.2	792	4.9	5.1	94	Biome 4
Huaypo	3540	13.7886	72.3158	17	8	1777.9	21.2	13.4	10.5	11.3	701	2.8	3.5	61	Biome 4
PLS‐2‐A	3533	13.8594	72.3825	3.5	7.9	2038	26.9	17	11.8	12.6	736	2.7	2.9	76	Biome 4
L.Vacas	3417	17.8308	65.6025	ND	ND	ND	10.7	ND	12.2	13.7	468	3.3	3.6	164	Biome 4
Marca Kotcha	3375	13.2539	72.3319	0.1	8.3	439.3	7.5	21.7	10.8	10.4	702	5.1	4.9	126	Biome 4
Condores	2885	7.63361	78.3736	ND	ND	ND	ND	ND	13	13.5	1107	4.1	6.9	39	Biome 2
Las Antennas	2604	0.5297	78.0186	1.3	7.5	163.3	25.5	18.9	15.1	15.4	1415	4.2	6.9	37	Biome 2
Erazo	2306	0.6019	77.9103	2.7	7.5	207	12.3	15.5	16.4	16.6	2307	5.7	6.9	90	Biome 2
Pomacochas	2100	6.45111	78.7986	ND	ND	ND	ND	ND	16.6	16.5	947	5.2	5.1	37	Biome 2
Consuelo	1360	13.9769	68.0503	10.5	5.7	35.4	78.7	23.3	20.5	21.3	2305	3.7	4	109	Biome 2
Pindo	1248	1.4867	78.3019	0.4	6	126.3	49.3	21	20.2	20.1	3993	3.2	8.6	114	Biome 2
Mera	1103	1.5986	78.165	0.4	6.4	161.3	26.5	20	20.5	20.5	4116	8.1	8.3	59	Biome 2
T.Hugo Orthiz	1051	1.4383	78.0172	2.3	6.6	190.3	21.5	24	20.8	20.7	4393	7.9	5.8	113	Biome 2
Landia road	1023	1.4569	78.0264	1.6	6.9	177.7	25.2	20.5	21	21.1	4421	4.1	9.3	78	Biome 2
Tendamina	1006	5.97	78.7636	ND	ND	ND	ND	ND	22	22.8	1206	8.5	4.3	120	Biome 3
San Ignau	161	14.9903	65.6622	2	8.9	58	5.9	28.2	24.9	26.5	1910	6.1	6.9	70	Biome 1
Loma Suarez	159	14.7642	64.9556	2	7.4	136	9.3	28.1	25.8	27	1862	10.5	11.8	39	Biome 1
P. de ibarre	157	14.8697	64.9758	2	7.3	131	7.1	33	25.7	27	1898	5.8	7.8	39	Biome 1
Laguna Azul	155	14.9872	64.815	2	6.7	30	3.8	26.1	25.7	27.1	1973	7.1	9.5	26	Biome 1
L.Belan	155	14.4569	64.8572	2	7.5	29	4.5	31.6	25.8	27	1763	8.7	9.5	46	Biome 1
L.Suarez	154	14.7642	64.9556	2	7.8	41	3.7	28.4	25.8	27.1	1915	5.1	7.8	25	Biome 1
Coitarama	150	14.5036	64.8703	2	8.1	29	0	27.6	25.8	27	1783	7.1	8	52	Biome 1

N° head capsules, the total number of chironomid head capsules retrieved from each lake; Biome 1, tropical/subtropical grass savanna‐shrubland; Biome 2, tropical/subtropical moist broadleaf forest; Biome 3, tropical/subtropical dry broadleaf forest; B4 = montane grass and shrub land.

### Chironomid and environmental variable analysis

**Figure 2:**
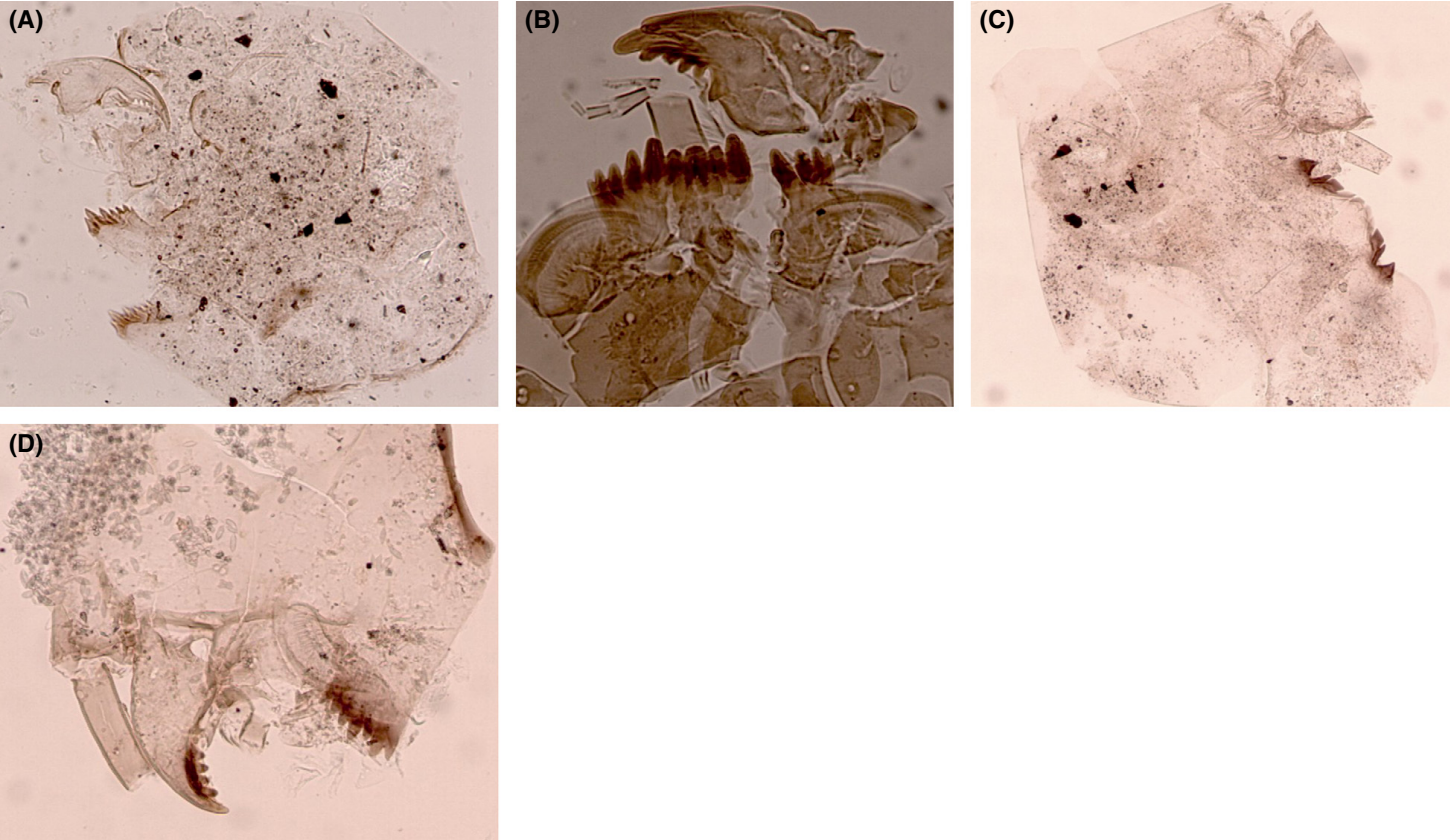
Subfossil larval remains of Chironomini from tropical South America. (A) Chironomini type I; (B) Chironomini type II; (C) *Cladopelma* type I; (D) *Reithia/Pseudochironomus*.

**Figure 3:**
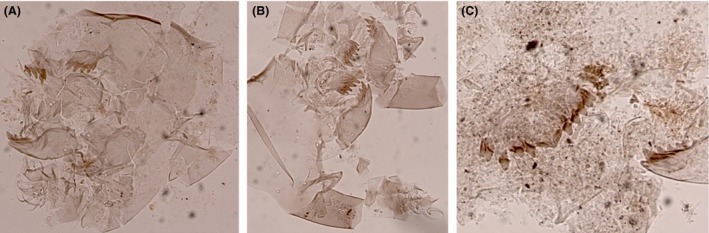
Subfossil larval remains of Tanytarsini from tropical South America. (A) *Tanytarsus* type I; (B) *Tanytarsus* type II; (C) *Tanytarsus* type III.

**Figure 4:**
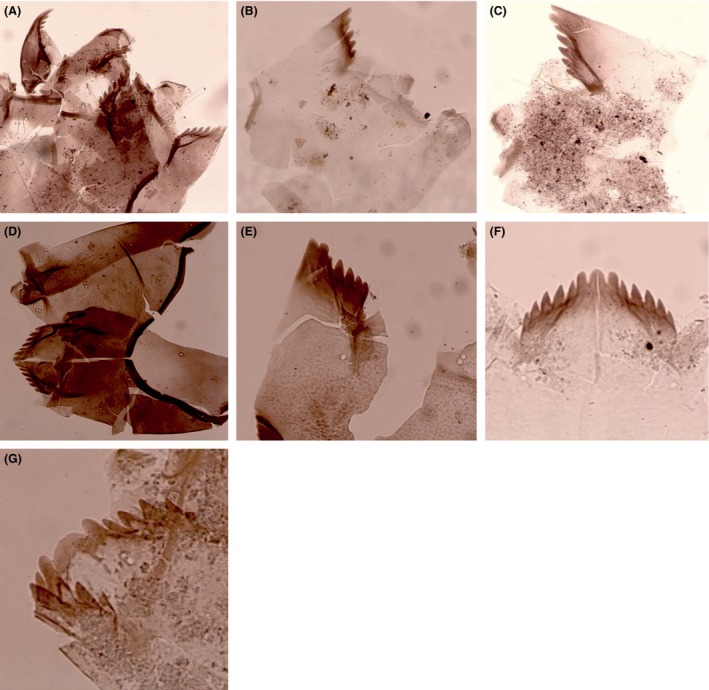
Subfossil larval remains of Orthocladiinae from tropical South America. (A) *Cricotopus/Paratrichocladius* type I; (B) *Cricotopus/Paratrichocladius* type II; (C) Cricotopus*/Paratrichocladius* type III; (D) *Cricotopus/Paratrichocladius* type IV; (E) *Cricotopus/Paratrichocladius* type V; (F) *Cricotopus/Paratrichocladius* type VI; (G) *Cricotopus/Paratrichocladius* type VII.

**Figure 5:**
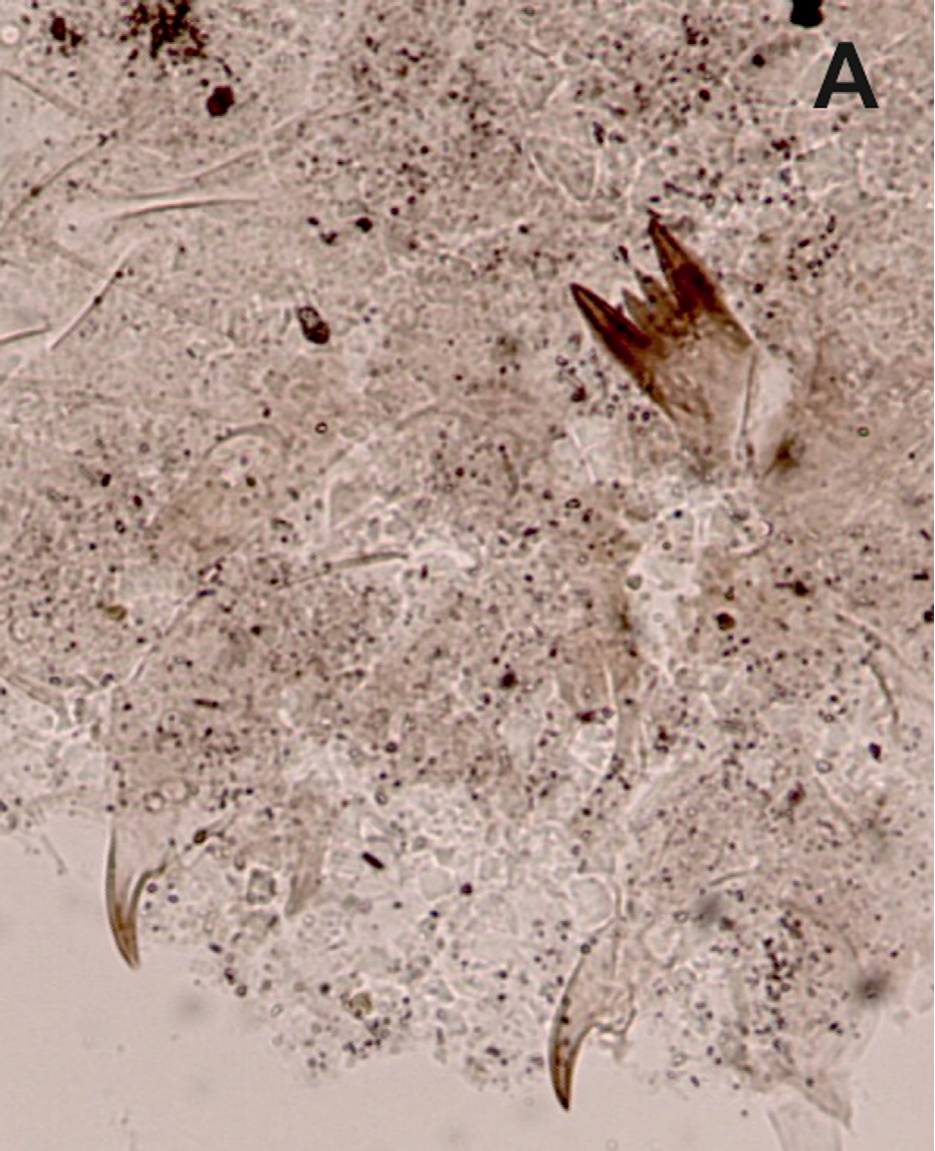
Subfossil larval remains of Tanypodinae from tropical South America. (A) Tanypodinae type I.

Surface sediment samples were collected from the deepest point of each lake to ensure representative samples of the entire chironomid fauna (Heggen et al. 2012). Depth was measured with a depth sounder. Sediments were collected using either a modified Livingston piston corer or a gravity corer. The uppermost centimeter (0–1 cm), which represents the most recent deposits, was analyzed. Preparation of samples followed standard methods (Brooks et al. 2007). Chironomids were identified to the highest possible taxonomic resolution under a compound light microscope at 200–400× magnifications. Because most Neotropical chironomid larvae cannot be reliably identified to species, specimens were assigned to morphotypes using references including Wiederholm (1983), Epler (2001) Rieradevall and Brooks (2001), Brooks et al. (2007), Cranston (2010), and several local taxonomic works including Prat et al. (2011), and Trivinho‐Strixino (2011). A list of all taxa identified can be found in Table 2 and descriptions of morphotypes not illustrated in the literature are provided (Figs. 2, 3, 4, 5).

**Table 2:** List of taxa found

Taxon	Occ	Max	<1000 (m a.s.l)	1000–3000 (m a.s.l)	>3000 (m a.s.l)
Chironominae					
*Beardius*	2	1	−	+	−
Chironomini type I	2	9	+	−	−
Chironomini type II	2	2	+	−	+
*Chironomus anthracinus*‐type	44	89	+	+	+
*Chironomus plumosus*‐type	10	51	+	+	+
*Cladopelma* ‘cf’ *laccophila*‐type	3	4	+	−	−
*Cladopelma* type I	4	1	+	−	−
Cladopelma ‘cf’ *lateralis*‐type	3	20	+	+	−
*Cladotanytarsus*	7	15	+	−	−
*Dicrotendipes*	1	11	−	+	−
*Einfeldia*	3	3	−	+	−
*Glyptotendipes*	3	5	−	+	−
*Goeldichironomus*	9	22	+	+	−
*Microchironomus*	7	15	+	+	−
*Micropsectra*	8	21	+	+	+
*Parachironomus*	3	4	−	−	+
*Paralauterborniella*	2	3	+	−	−
*Paratanytarsus*	30	70	−	+	+
*Phaenopsectra*	5	4	−	+	−
*Polypedilum nuberculosum*‐type	14	40	−	−	+
*Polypedilum nubifer*‐type	9	37	+	+	+
*Polypedilum sordens*‐type	1	1	+	−	−
*Reithia*/*Pseudochironomus*	7	50	−	+	+
*Rheotanytarsus*	4	2	−	+	+
*Stempellina*	1	1	+	−	−
*Tanytarsus* type I	5	18	−	+	−
*Tanytarsus* type II	12	52	−	+	+
*Tanytarsus* type III	4	4	+	−	−
*Zavreliella*	1	2	+	−	−
Orthocladiinae					
*Corynoneura* ‘cf’ *coronata*‐type	5	16	−	+	−
*Corynoneura* ‘cf’ *lobata*‐type	1	2	+	−	−
*Cricotopus*/*Paratrichocladius* type I	15	48	−	−	+
*Cricotopus*/*Paratrichocladius* type II	21	46	−	+	+
*Cricotopus*/*Paratrichocladius* type III	1	9	−	+	+
*Cricotopus*/*Paratrichocladius* type IV	1	9	−	−	+
*Cricotopus*/*Paratrichocladius* type V	10	41	−	−	+
*Cricotopus*/*Paratrichocladius* type VI	1	8	−	−	+
*Cricotopus*/*Paratrichocladius* type VII	11	75	−	−	+
*Limnophyes*	16	8	+	+	+
*Paracricotopus*	2	2	−	+	+
*Parakiefferiella*	1	2	−	+	−
*Parametriocnemus*	6	7	−	+	+
*Pseudorthocladius*	6	4	−	+	+
*Pseudosmittia*	33	67	−	+	+
*Smittia*	2	2	−	+	−
*Thienemanniella*	2	1	−	−	+
*Thienemanniella* ‘cf’ *clavicornis*‐type	2	1	−	−	+
Tanypodinae					
*Guttipelopia*	1	3	−	+	−
*Labrundinia*	2	6	+	+	−
*Larsia*	1	1	−	+	−
*Procladius*	15	57	+	+	+
*Psectrotanypus*	1	1	−	+	−
Tanypodinae I	2	4	+	−	−
*Tanypus*	3	8	+	−	−
Diamesinae	2	4	−	+	−

Occ, number of lakes within which the taxon occurs; Max, maximum number of individuals found in any one site; +, The occurrence of taxa across three broad elevational boundaries (<1000 m a.s.l., 1000–3000 m a.s.l, and >3000 m a.s.l).

Measurements of pH, conductivity, and water temperature (WT) were made at each lake at the time of sediment sampling. Three measurements were recorded 0.5 m below the water surface and then averaged. The organic content of the sediment was established through loss‐on‐ignition, and the results are expressed as percentage weight loss of dry sediment after burning at 550°C for 4 h (Heiri et al. 2001). Local meteorological data were not available for all the study sites, and so climatic variables were obtained from the WORLDCLIM database (Hijmans et al. 2005). The data are a compilation of monthly averages for at least ten years but in most cases 30–50 years between 1960 and 1990 or 1950 and 2000 at a 1‐km^2^ resolution. At six lakes (Lagunas Vacas, Larrati, Patos, Pomacochas, Tendamina, and Condores), depth, pH, conductivity, and LOI were not recorded due to logistical limitations. These sites were omitted from the multivariate analysis. For a summary of all variables, see Table 1.

### Exploratory statistics

Detrended correspondence analysis (DCA) was initially used as an indirect ordination method to assess the gradient lengths in compositional units of taxon turnover (Hill and Gauch 1980). The gradient length of DCA axis 1 was 5.2 standard deviation units (SD), which suggests a unimodal response, and that linear ordination methods were not appropriate (Ter Braak 1987).

To assess the relationships between chironomid assemblages and the environmental variables, canonical correspondence analysis (CCA) was used. Analysis was performed on percentage square‐root‐transformed species assemblage data and rare taxa were down weighted, and no species were removed from the analysis. Canonical correspondence analysis, using single and partialed‐out variables, was used in order to assess how much of the variance in the species data is described by any individual variable. The ratio of *λ*1/*λ*2 (i.e., the ratio of eigenvalues of the first constrained CCA axis and second unconstrained CA axis) was used as a measure of how important the variable of interest is as an ecological determinant (Ter Braak 1987).

Elevation is a dominant feature of Andean environments and exerts a strong influence on many variables, as a result many covary and problems of latent variables and collinearity arise. Covariance between temperature and elevation, for example, would mask the influence of either one. Removing covarying variables allows for a greater understanding of which variables were most important in describing species distributions. Highly correlated variables can be partialed out by analysis of the variance of their regression coefficients indicated by their Variance inflation factor (VIF; Leps and Smilauer 2003). Typically, VIFs >10 are considered as being highly correlated, and commonly used as the threshold above which variables should be excluded. O'Brien (2007), however, demonstrated that arbitrary thresholds for excluding variables can lead to problems more serious than the original collinearity. VIFs of 10, 20, 40, or higher do not in themselves call for the exclusion of a variable (O'Brien 2007). Here, we chose a more conservative deletion criteria for environmental variables than the common value of 10. Variables with a high VIF were systematically removed from the environmental variable dataset until the remaining variables had a VIF below 20. This process reduced the dataset to nine explanatory variables to be used in the CCA. The included variables are MAT, mean July temperature (MJT), MAP, WT, conductivity, depth, LOI, pH, and latitude. Elevation and longitude were excluded from the analysis.

For chironomid larval head capsule studies, generally, a minimum total head capsule count of 50 is advised (Heiri and Lotter 2001; Quinlan and Smol 2001). Rarefaction was used to estimate taxon richness across the dataset, as the number of chironomid larval head capsules retrieved from each lake was not uniform and was below the recommended minimum (Table 1). Rarefaction estimates species richness from random subsamples of a defined size (Hurlbert 1971). A minimum of 23 head capsules was used to represent the smallest number of chironomids extracted from any one lake. To assess the effect of incomplete sampling on richness estimates (i.e., not all taxa at a site being represented by the sampling), the abundance‐based coverage estimator (ACE) was used as a second measure of richness (Chao and Lee 1992; Chao et al. 2000). Coverage estimators recognize widespread abundant taxa are likely to be present in many samples so, instead, species occurring in <10 lakes are used to estimate richness (Magurran 2006). Simpson 1/D, divided by the number of species, was used as an independent measure of evenness to separate the signal from the richness estimates (Magurran 2006). Analyses were performed in R, using the package Vegan (Oksanen et al. 2013), Rioja (Juggins 2015) and CANOCO 5.0 (Ter Braak and Smilauer 2002).

## Results

### Taxonomic notes

The modern chironomid assemblages of the lakes we surveyed included several taxa for which we could find no descriptions in the regional chironomid taxonomic literature. Below, we have given these taxa informal names and provided descriptions of the diagnostic characters.

### Chironomini (Fig. 2A–D)

#### Chironomini type I (Fig. 2A)

##### Diagnostic features

Mentum with two short median teeth; six lateral teeth, 1st lateral tooth taller than median and outer lateral teeth. Mandible strongly curved with long apical tooth and four inner teeth. The taxon occurs at low elevations (<1000 m a.s.l.) and is present in two study sites.

#### Chironomini type II (Fig. 2B)

##### Diagnostic feature

Trifid median tooth with five lateral teeth. First lateral tooth at least twice as long as three median teeth. Ventromental plates are strongly curved and striated with a dark band which narrows and curves along the leading edge. Mandible with three inner teeth, apical tooth short twice the width of inner teeth. The taxon occurs at low elevations (<1000 m a.s.l.) and present in two study sites.

#### *Cladopelma* type I (Fig. 2C)

##### Diagnostic features

Mentum with paired, broad median teeth, five lateral teeth, 2nd lateral tooth flattened apically and twice as broad as 1st and 3rd lateral teeth, prominent gap between 3rd and 4th lateral tooth, 4th lateral tooth broad and acutely pointed at apex, outermost lateral tooth minute and fused to 4th tooth. Ventromental plates faintly striated, at least as broad as width of mentum. The arrangement of teeth on the mentum is similar to *Cladopelma* although this genus usually has seven lateral teeth. No mandibles were found. *Cladopelma* type I occurs at low elevations (<1000 m a.s.l.) and is present in four study sites. Cranston (2010) does not list *Cladopelma* from the Neotropics, although Wu et al. (2014) have found it in modern lake sediments in Central America.

#### *Reithia/Pseudochironomus* (Fig. 2D)

##### Diagnostic features

Single median tooth, six lateral teeth, 2nd lateral tooth strongly reduced. Large, curved narrow, strongly striated ventromental plates. Mandible with four inner teeth. Pedestal, without short spur. Larvae of *Reithia* are very similar to *Pseudochironomus* which are currently indistinguishable in the larval stages, both genera are known from the Neotropics (Trivinho‐strixino et al. 2009; Cranston 2010). *Reithia* is known from modern lake surface sediments in Patagonia (Massaferro et al. 2014) and Costa Rica (Wu et al. 2014). For clarity, we refer to the taxon as *Reithia/Pseudochironomus*. We record the taxon in seven mid‐ to high‐elevation lakes (>1000 m a.s.l.).

### Tanytarsini (Fig. 3 A–C)

#### Tanytarsus

*Tanytarsus* is a eurytopic genus with a worldwide distribution (Cranston 2010), and we identify three morphotypes of *Tanytarsus* (*Tanytarsus* type I, *Tanytarsus* type II, and *Tanytarsus* type III) distinguishable from *Paratanytarsus* and *Micropsectra* by the presence of three teeth on the premandible. Massaferro et al. (2013) described eight morphotypes of Tanytarsini from Patagonia. Were possible, we compare the morphotypes described here with those descriptions from Patagonia.

##### Diagnostic features

All morphotypes have a single median tooth with five lateral teeth. *Tanytarsus* type I (Fig. 3A) is distinct from the other two morphotypes by the possession of a short, pointed spur on the antennal pedestal. The antennal pedestal is about 1.5 times as long as broad. There are two inner teeth on the mandible. The taxon is found at mid‐elevation (1000–3000 m a.s.l.) and recorded in five lakes. The taxon most resembles morphotypes *Tanytarsus* 1A described by Massaferro et al. (2013) from Patagonia. In Patagonia, the taxon is considered cosmopolitan occurring in many habitats across the region. In *Tanytarsus* type II (Fig. 3B), the antennal pedestal is about twice as long as broad and lacks a spur. The mandible has three inner teeth, but the lower inner tooth is small and is not completely separate from the mandible. The taxon most resembles morphotypes *Tanytarsus* D from Patagonia, a rare taxon only found in Northern Argentina and Chile (Massaferro et al. 2013). *Tanytarsus* type II is found in mid‐ to high‐elevation lakes (>1000 m a.s.l.) occurring in 12 lakes. *Tanytarsus* type III (Fig. 3C) has a distinctive large triangular median tooth on the mentum, and the pedestal is 1.5 times as long as broad with no pedestal. The mandible has three inner teeth. The taxon is only found in low‐elevation lakes (<1000 m a.s.l.), and no comparable taxon was described in Patagonia.

### Orthocladiinae (Fig. 4A–G)

#### *Cricotopus*/*Paratrichocladius*

Larvae of *Cricotopus* cannot be separated with certainty from those of *Paratrichocladius*. Seven morphotypes were identified in our material and were distinguished on the basis of mentum morphology. Two of these taxa were previously referred to as *Cricotopus/Orthocladius* type I and type II by Williams et al. (2012), but at present *Orthocladius* is not known to occur in the Neotropics (Cranston 2010). All the taxa occur in high‐elevation lakes (>3000 m a.s.l.) although *Cricotopus/Paratrichocladius* type I and type II also occur in lakes between 1000 and 3000 m a.s.l.

##### Diagnostic features

All taxa have a single median tooth and six lateral teeth. *Cricotopus/Paratrichocladius* type I (Fig. 4A) has a long dark ventromental plate and the head capsule is usually dark. In *Cricotopus/Paratrichocladius* type II (Fig. 4B), the mentum has a curved concave profile, as the 2nd lateral tooth is very short and there is a wide gap between the 2nd and 3rd lateral teeth. In *Cricotopus/Paratrichocladius* type III (Fig. 4C), the end of the ventromental plate is strongly curved upwards, and the 2nd lateral tooth is narrower than the 1st lateral tooth but taller than the 3rd lateral tooth. The head capsule of *Cricotopus/Paratrichocladius* type IV (Fig. 4D) is heavily pigmented with lateral teeth decreasing gradually in size. The median tooth is about as broad as the first lateral tooth; in other taxa in this genus group, the median tooth is about twice as broad as the first lateral tooth. The 2nd lateral tooth is hardly reduced and is positioned midway between the 1st and 3rd lateral tooth. In other taxa in this genus group, the 2nd lateral tooth is positioned closer to the 1st than the 3rd lateral tooth. *Cricotopus/Paratrichocladius* type V (Fig. 4E) is similar to *Cricotopus/Paratrichocladius* type II which, however, can be distinguished by the ventromental plate that bulges beyond the mentum. *Cricotopus/Paratrichocladius* type VI (Fig. 4F) is similar to *Cricotopus/Paratrichocladius* type IV; however, the mentum is less pigmented and the 2nd lateral tooth is reduced and positioned closer to the 1st lateral than the 3rd lateral tooth. *Cricotopus/Paratrichocladius* type VII (Fig. 4G) has a broad, rounded median tooth, which is about three times the width of the first lateral tooth. The 1st lateral tooth is taller than the median tooth.

### Tanypodinae (Fig. 5A)

One taxon of Tanypodinae could not be allocated to a known genus. In Tanypodinae type I, the ligula has two large outer and two small inner teeth. Tanypodinae type I occurred in two lakes at low elevations (<1000 m a.s.l.).

### Chironomid distribution

In total, 4587 individual chironomid larval head capsules were obtained from the sediments of the 59 lakes. The dataset consists of 55 taxa: 29 Chironominae, 18 Orthocladiinae, seven Tanypodinae, and one Diamesinae (Table 2).

**Figure 6:**
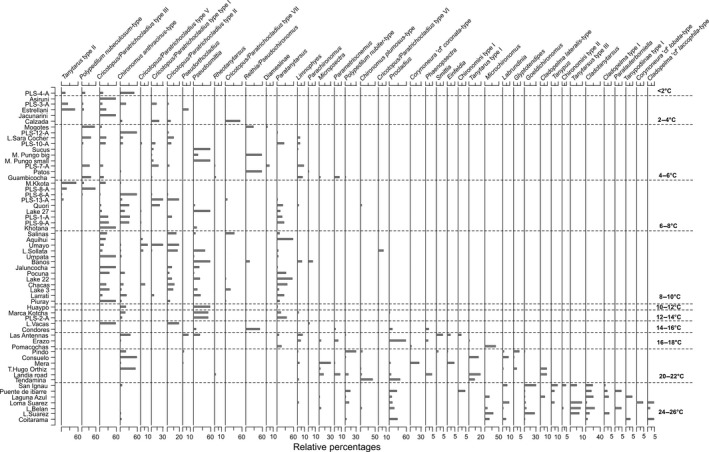
Chironomid assemblages for the 59 lakes. Lakes are ordered by mean annual temperature from cold to warm. Lakes are separated every 2°C along the temperature gradient with chironomid taxa shown in order of occurrence. Only taxa present in more than two lakes are shown.

**Figure 7:**
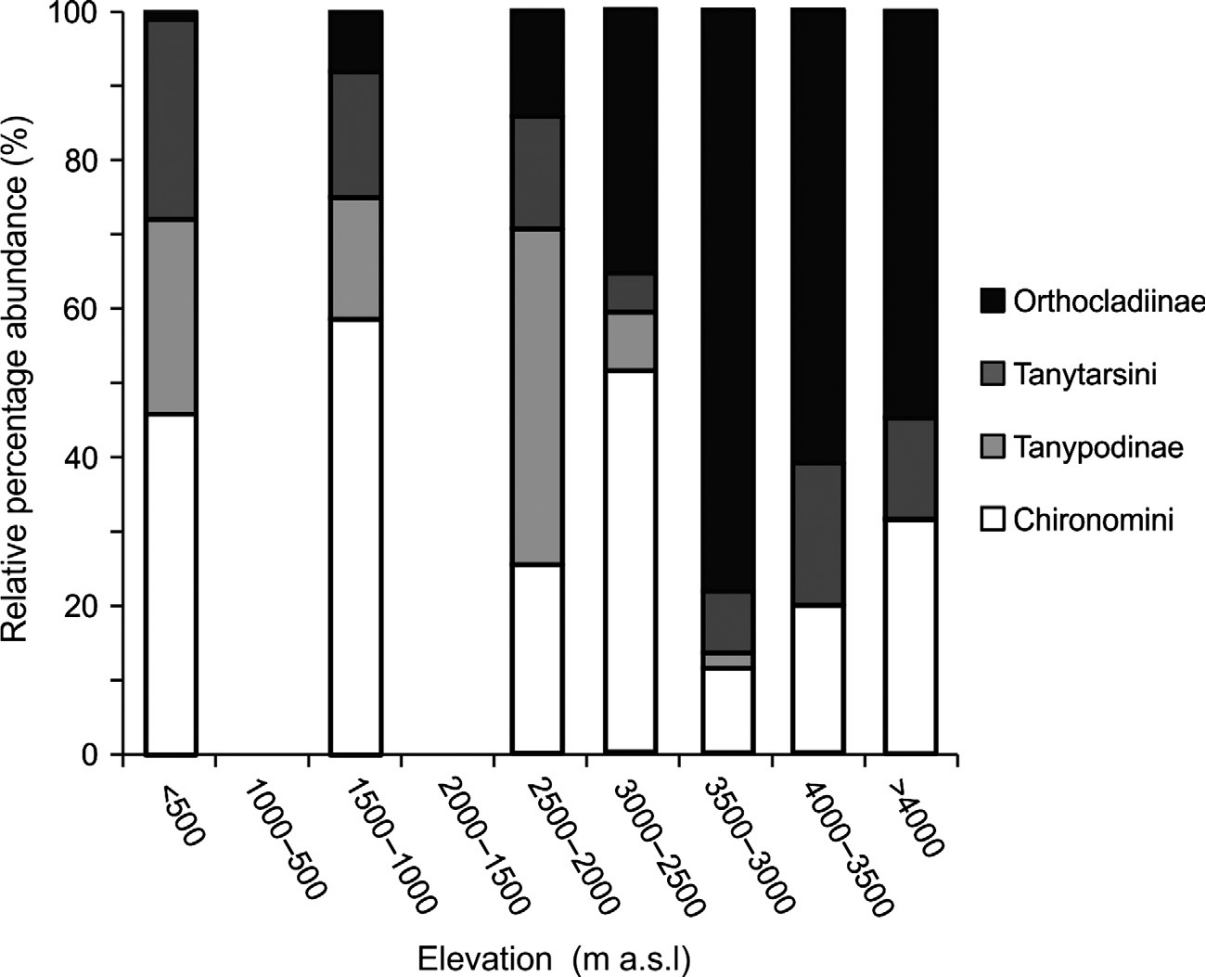
Relative percentage of the chironomid assemblages divided by subfamily (Orthocladiinae, Tanypodinae, and Chironominae), Chironominae is further divided into two tribes, Tanytarsini and Chironomini. The lakes are grouped into elevation bins of 500 m up to lakes >4000 m a.s.l.

*Chironomus anthracinus* type and *Limnophyes* were the most widespread taxa, occurring in lakes across all biomes and the entire elevation range (Figs. 6 and 7). Twenty‐seven taxa occurred in the seven (<200 m.a.s.l.) lowland Amazonian lakes, 14 taxa, including *Cladotanytarsus*,* Paralauterborniella,* and *Tanypus*, were restricted to these localities. Thirty‐seven taxa occurred at mid‐elevations within the tropical and subtropical moist/dry broadleaf forest (1000–3000 m.a.s.l.). Seventeen of these, including *Tanytarsus* type I*,* and *Corynoneura* ‘cf’ *coronata*‐type, were restricted to these lakes. Twenty‐eight taxa were identified from high‐elevation sites (>3000 m.a.s.l.), 11 of which were unique to these localities, including *Parachironomus, Reithia/Pseudochironomus, Tanytarsus* type II, and *Cricotopus/Paratrichocladius* type II. At high elevations (>3000 m a.s.l.), there are clear faunal changes along the latitudinal gradient, *Parametriocnemus* was in greatest abundance at low latitudes (<2°S), and further south taxa, such as *Pseudosmittia* and morphotypes of *Cricotopus/Paratrichocladius*, dominated the assemblage.

Above 3000 m a.s.l., Orthocladiinae is the most important subfamily, representing over 50% of the chironomid fauna in high‐elevation lakes with Chironomini and Tanytarsini making up minor components (Fig. 7). Below 3000 m a.s.l., the dominance of Orthocladiinae declines, and is replaced by Chironomini. In lowland Amazonian lakes, Orthocladiinae is a minor component of the chironomid fauna, making up <5% of assemblages. Tanypodinae, Tanytarsini, and Chironomini dominate low‐elevation lakes.

**Figure 8:**
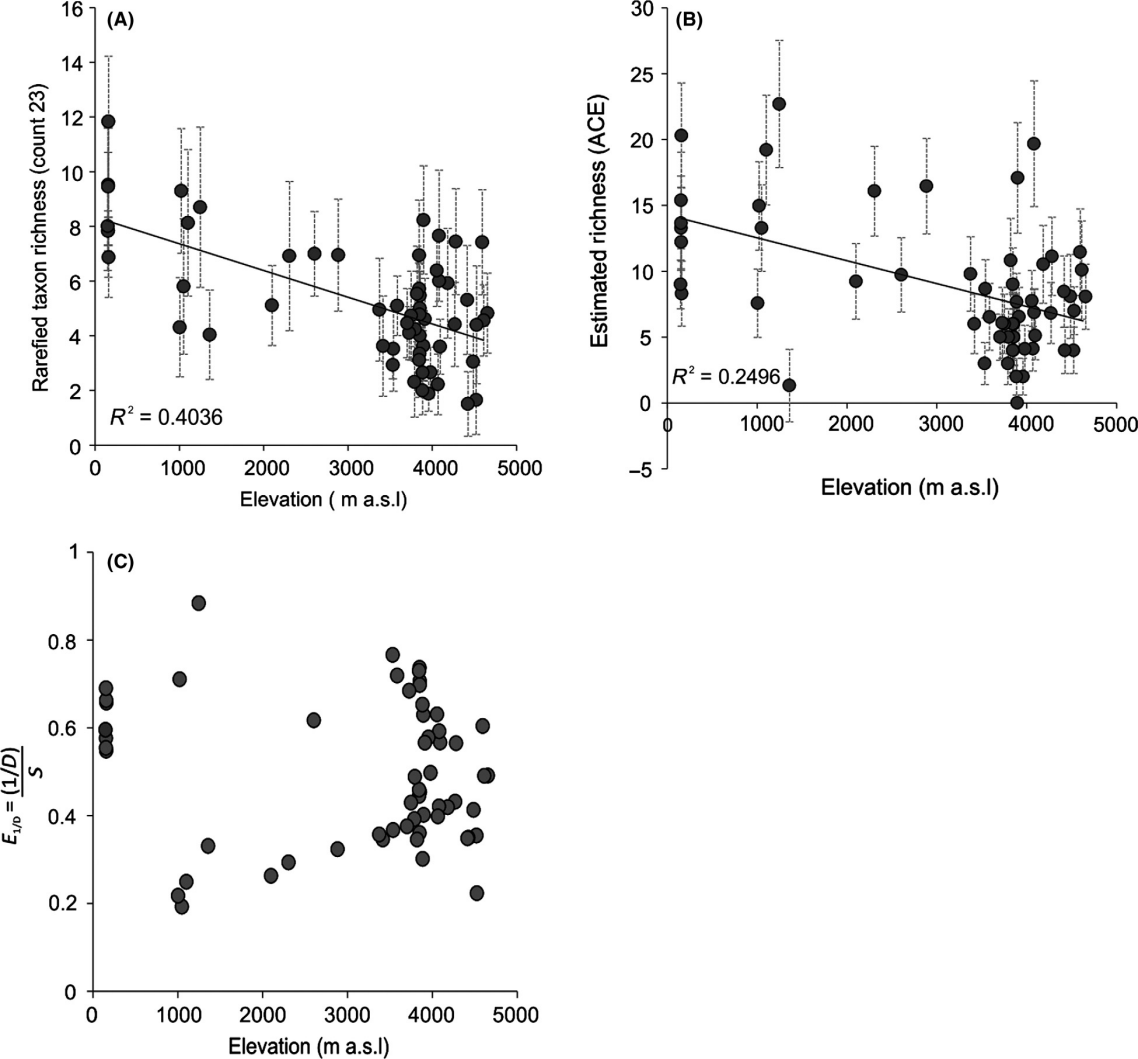
Chironomid taxon richness and evenness. (A) Rarefied taxon richness was estimated using count sizes of 23 (minimum count in the dataset). (B) Richness estimator (ACE) using rare individuals only (occurred in <10 lakes). (C) Simpson 1/D divided by the number of species was used as an independent measure of evenness to separate the signal from the richness estimates. Sample errors are 95% confidence intervals.

Taxon richness varied between 11.8 and 1.5 with a mean value of 5.4. Maximum richness occurred at low elevations (<200 m a.s.l.) and minimum richness occurred at high elevations (> 3000 m a.s.l.) (Fig. 8). Taxon richness in high Andean sites was generally lower than that found in Amazonian sites. At mid‐elevations (1000–3000 m a.s.l.), taxon richness was greater than the minimum values recorded above 3000 m a.s.l. (Fig. 8A). The ACE suggested considerable variability in richness estimates. Most high‐elevation sites were at the lower end of the richness scale, with the exception of two lakes lying higher than 3000 m a.s.l. Contrary to the rarefied estimates, peak richness (ACE) occurred at *c*. 1000 m a.s.l. (Fig. 8B). Overall, the relationship between richness and elevation is not significant. There are no clear trends of evenness changing with elevation, both the most and least even sites occur at *c*. 1000 m a.s.l. (Fig. 8C).

### Ordination

**Figure 9:**
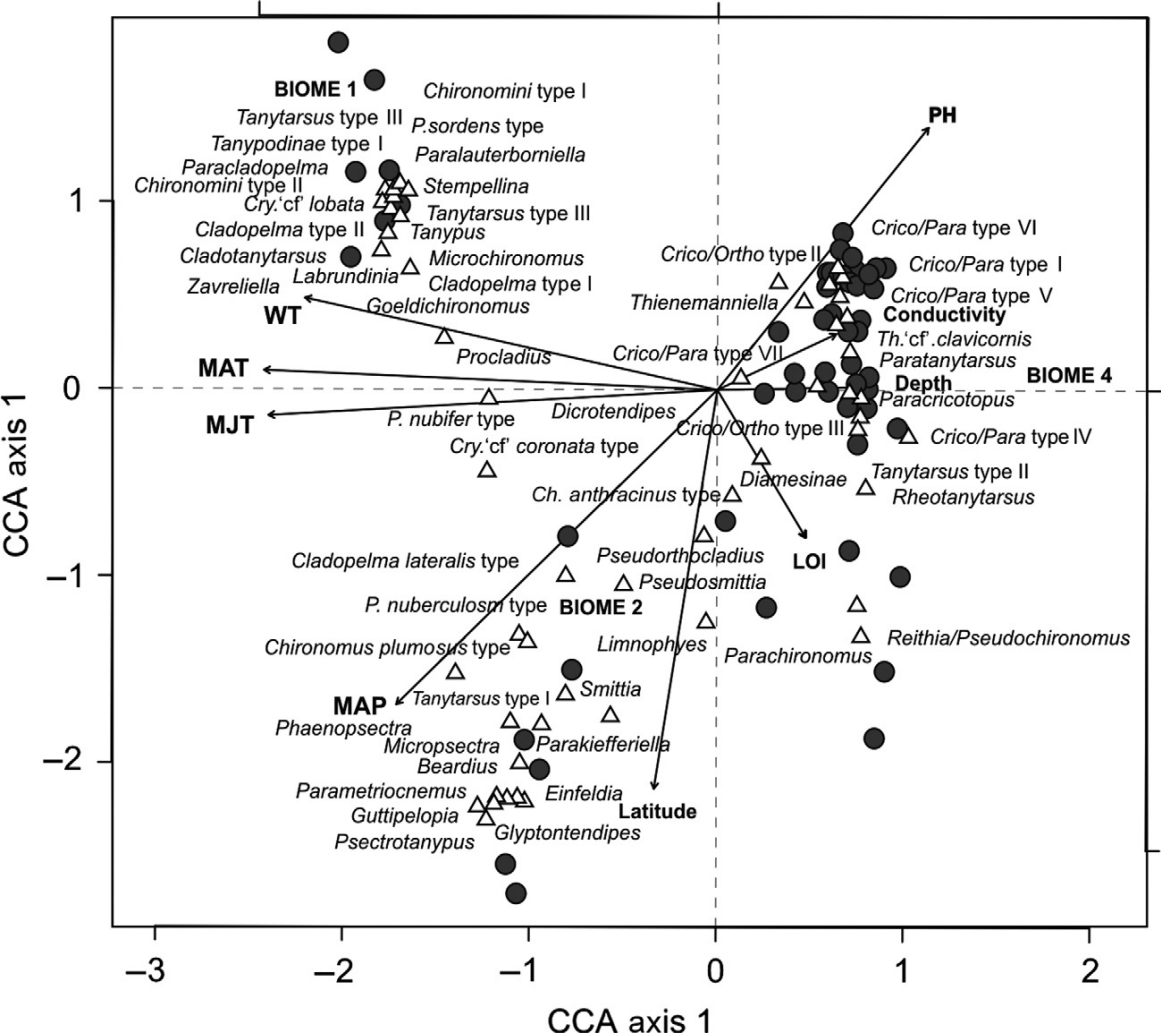
Canonical correspondence analysis (CCA) biplot of the nine explanatory variables once those with a VIF >20 were removed (i.e., Elevation and Longitude), study sites and chironomid taxa. Circles denote study sites; white triangles indicate the location of taxa. Biomes have been included as nominal variables. B1 = tropical/subtropical grass savannah‐shrubland, B2 = tropical/subtropical moist broadleaf forest, B4 = montane grass and shrub land. Only one lake occurs in B3 (tropical/subtropical dry broadleaf forest) and so its occurrence within the ordination space cannot be inferred, Biome 3 is excluded as a nominal variable.

The subset of explanatory variables used in a CCA explained a total of 34.03% of the variance in the species assemblage data (Table 3). The first two CCA axis account for 61.7% of the explained variance (*λ*_1_ = 0.792, *λ*_2_ = 0.466). Temperature variables (MAT, MJT, and WT) correlate with axis 1, while latitude correlates with axis 2 (Fig. 9). Three variables, LOI, depth, and conductivity, were not significant. Conductivity and pH were inversely correlated with MAP. Lake assemblages clustered within three broad groups. All lowland Amazonia lakes (Biome 1) plot as a tight cluster in the top left of Figure 9, associated with high air and WT. Lakes above 4000 m a.s.l. had high pH and conductivity and cool temperatures and plot on the right of the CCA plot (Biome 4). The high‐elevation sites are spread out associated with latitude; the chironomid fauna of northern sites of Ecuador is distinct from that of the higher latitude lakes of Peru and Bolivia. Mid‐elevation lakes (c. 1000–4000 m a.s.l.) within the tropical and subtropical moist/dry broadleaf forests (Biome 2) plot in the bottom left, and the chironomid assemblages were more variable but mostly associated with high precipitation, low pH, and low conductivity. Low‐latitude Ecuadorian sites grouped with high MAP. These lakes receive a higher annual precipitation (*c*. 2585 mm/year) compared with lakes at higher latitudes in Peru and Bolivia (*c*. 958 mm/year).

**Table 3:** Summary of the canonical correspondence analysis (CCA) using constraining variables. The variables included: mean annual temperature (MAT), mean January temperature (MJT), water temperature (WT), mean annual precipitation (MAP), pH, loss‐on‐ignition (LOI), conductivity, and depth. The significance of the constraining axis and each environmental variable was tested using an ANOVA (999 permutations). The t‐values for each variable were obtained by refitting the results of the constrained ordination as a multiple response linear model

	Inertia	Proportion										
Total	6	1										
Constrained	2.042	0.34										
Unconstrained	3.959	0.66										

	CCA1	CCA2	CCA3	CCA4	CCA5	CCA6	CCA7	CCA8	CCA9	Residual		
Eigenvalue	0.792	0.466	0.276	0.168	0.118	0.08	0.057	0.049	0.034			
Proportion Exp.	0.388	0.228	0.135	0.082	0.06	0.09	0.03	0.024	0.017			
Cumulative Pro.	0.388	0.617	0.751	0.834	0.892	0.93	0.96	0.983	1			
Chi Square	0.7924	0.4663	0.2761	0.1684	0.1182	0.0792	0.0572	0.0492	0.0346	3.9588		
Pseudo‐*f*	8.807	5.183	3.068	1.872	1.314	0.88	0.636	0.547	0.3845			
*P*	0.001	0.001	0.001	0.011	0.199	0.774	0.972	0.982	0.999			

	Canonical coefficients				*t*‐values				Interset correlation			
	CCA1	CCA2	CCA3	CCA4	CCA1	CCA2	CCA3	CCA4	CCA1	CCA2	CCA3	CCA4
MAT	−0.987	0.041	0.012	0.097	−3.691	0.816	−0.871	3.814	−0.966	0.039	0.008	−0.07
MJT	−0.977	−0.057	−0.033	−0.145	2.253	−0.173	0.643	−3.837	−0.956	−0.054	−0.028	−0.104
MAP	−0.697	−0.686	0.172	−0.096	−1.689	−7.062	6.335	−1.215	−0.682	−0.648	0.136	−0.069
WT	−0.898	0.198	−0.049	−0.157	−0.993	1.274	−0.857	−1.107	−0.879	0.188	−0.041	−0.113
pH	0.458	0.571	0.136	−0.447	1.793	2.145	1.963	−3.565	0.448	0.54	0.12	−0.318
LOI	0.193	−0.32	0.453	0.209	2.586	−0.381	2.539	0.749	0.189	−0.303	0.363	0.149
Depth	0.316	0.007	0.039	0.265	−1.36	1.119	1.395	−0.385	0.309	0.006	0.031	0.189
Conductivity	0.259	0.122	0.098	−0.475	1.093	−0.548	0.295	−2.442	0.253	0.115	0.079	−0.338

	Chi squre	Pseudo‐*f*	*P*									
MAT	0.235	2.616	0.003									
MJT	0.164	1.819	0.03									
MAP	0.218	2.422	0.004									
WT	0.474	5.268	0.001									
pH	0.405	4.505	0.001									
LOI	0.18	2.007	0.026									
Depth	0.146	1.627	0.11									
Conductivity	0.087	0.964	0.533									

The ordination effectively distinguishes warm‐tolerant (e.g., *Cladopelma* type I, *Tanypus, Stempellina*) and cold‐tolerant taxa (e.g., *Tanytarsus* type II, *Thienemanniella* ‘cf’*clavicornis‐*type*)* and taxa correlating with high precipitation and low latitude (e.g., *Smittia* and *Einfeldia*). Canonical correspondence analysis using single constraining variables (Table 4) reveals that climate is the most important ecological determinant of the variance in the chironomid assemblages. Climate parameters (MAT, MJT, and MAP) and WT, when used as single constraining variables, explain over 10% of the variance in the chironomid dataset. Mean annual temperature performs best as a single constraining variable (*λ*_1_/*λ*_2_ = 1.4310; variance explained = 12.93%).

**Table 4:** Summary of the canonical correspondence analysis (CCA) using single constraining variables and partialed‐out variables. The ratio of the first constrained and second unconstrained eigenvalues (*λ*1:*λ*2), variance explained, and significance level (999 unrestricted permutations) are shown. Variables are mean annual temperature (MAT), mean January temperature (MJT), water temperature (WT), mean annual precipitation (MAP), pH, loss‐on‐ignition (LOI), conductivity, and depth

Variable	Covariables	λ1/λ2	Variance explained (%)	*P*
MAT	None	1.431	12.93	0.001
MAT	MJT, MAP, WT, pH, LOI, Depth, Conductivity, Latitude	1.3032	11.9	0.001
MJT	None	1.4	12.73	0.001
MJT	MAT, MAP, WT, pH, LOI, Depth, Conductivity, Latitude	1.2651	11.54	0.001
WT	None	1.23	11.21	0.001
WT	MJT, MAT, MAP, pH, LOI, Depth, Conductivity, Latitude	1.115	10.13	0.001
MAP	None	0.9	10.3	0.001
MAP	MJT, MAT, WT, pH, LOI, Depth, Conductivity, Latitude	0.84	9.49	0.001
Latitude	None	0.5034	7	0.001
Latitude	MJT, MAT, MAP, WT, pH, LOI, Depth, Conductivity	0.544	6.95	0.001
pH	None	0.5	6.23	0.001
pH	MJT, MAT, MAP, WT, LOI, Depth, Conductivity, Latitude	0.5903	6.8	0.001
LOI	None	0.2393	3.23	0.062
LOI	MJT, MAT, MAP, WT, pH, Depth, Conductivity, Latitude	0.238	3	0.102
Depth	None	0.1904	2.44	0.24
Depth	MJT, MAT, MAP, WT, pH, LOI, Conductivity, Latitude	0.2595	3	0.057
Conductivity	None	0.1792	2.34	0.296
Conductivity	MJT, MAT, MAP, WT, pH, LOI, Depth, Latitude	0.2012	2.4	0.272

## Discussion

### Richness

Our data show the taxon richness of chironomids across a large geographic region from the tropical Andes to lowland Amazonia, covering an altitudinal gradient of 4505 m and a MAT gradient of *c*. 25°C from 0.8 to 25.8°C. The dataset includes four biomes that are largely governed by climatic parameters associated with changing elevation. Lowland Amazonian lakes (<500 m a.s.l.) generally have richer faunas than high‐elevation lakes (Fig. 8). Lowland Amazonian lakes in the present study also have the highest proportion of unique taxa (*c*. 51% of taxa identified in Amazonian lakes were only recorded in these localities). Changes in diversity associated with elevation have been noted in other chironomid studies in the tropics. Working in the Yucatan peninsula, Pérez et al. (2013) found just 15 chironomid taxa at high elevations and 51 at low elevations (0–1560 m a.s.l.). In tropical East Africa, Eggermont et al. (2010) found a diverse overall assemblage of 81 taxa in 65 lakes over a similar climatic and altitudinal gradient (MAT range: 2.1–28.1°C, and 489–4575 m a.s.l.). Chironomid species diversity at high elevations (6 taxa on average; N2 = 3.2) was less than in low‐ to mid‐elevation lakes (15 taxa on average; N2 = 5.5) (Eggermont and Verschuren 2007). Jacobsen et al. (1997) in a study of a low‐elevation Ecuadorian stream also found aquatic insect richness increased with streams temperature. The strongest relationship between the measured environmental variables and increased species richness within the current dataset was with decreasing elevation and rising temperatures. Our data support other tropical chironomid studies and suggest chironomid species richness is generally higher in lakes within regions of higher ambient temperature; however, more data from mid‐ to low‐elevation lakes are needed to verify this relationship.

### Environmental controls

Over a range of spatial scales, from continental to within habitat, chironomids have been shown to respond to many different environmental drivers; these include temperature (Jacobsen 2004; Siqueira et al. 2008; Roque et al. 2010b; Eggermont and Heiri 2011), depth (Chen et al. 2013), salinity (Eggermont et al. 2006), hypolimnetic oxygen and phosphorus (Little and Smol 2001; Ramírez and Pringle 2006), pH (Nyman et al. 2005), and macrophytes (Brodersen et al. 2001). In our study, we were unable to measure DO and macrophyte density or diversity, and therefore, our knowledge of the drivers of chironomid distribution remains incomplete. Nevertheless, 34.03% of taxon variance can be explained by the subset of nine explanatory environmental variables we measured. The remaining *c*. 66% of variance not explained is likely a result of the influence of important unmeasured variables (e.g., dissolved oxygen), biotic interactions, or perhaps stochastic effects (ter Braak 1987). Of those variables measured, we have demonstrated that climate (MAT and MAP) predominantly controls the variation in species composition.

### Temperature

Many taxa are distributed along the CCA axis 1 temperature gradient, which is strongly associated with elevation. The strong relationship between temperature and distribution of chironomid assemblages has been documented in many other areas of the world (*see reviews in* Eggermont and Heiri 2011). Temperature is one of the most influential environmental variables, particularly for aquatic insects (Danks 2007), as it plays a key role in controlling metabolism (Lencioni et al. 2008), growth and development (Mckie et al. 2004), voltinism (Benke 1998), and behavior (Armitage 1995). Our data reflect trends apparent in other tropical chironomid datasets. Eggermont et al. (2010) identified MAT as a dominant variable in explaining chironomid species distributions in the Afrotropics. As in our study, species of the genus *Cladotanytarsus* were found in greatest numbers in low‐elevation warm tropical African lakes. Massaferro and Larocque (2013) and Massaferro et al. (2014), who studied subfossil chironomids in lakes across extra‐tropical Patagonia, also showed that chironomid species distribution in southern South America is determined by air temperature. In Patagonian lakes, *Phaenopsectra* was found to inhabit temperature ranges between 3°C and 16°C and was most abundant between *c*. 14 and 16°C (Massaferro and Larocque 2013; Massaferro et al. 2014). In the current tropical Andean dataset, this taxon is also found in relatively warm habitats (*c*. 12–22°C) but is absent from cooler lakes.

Many of the taxa identified in the tropical Andes also occur in extra‐tropical Patagonia, but the variables controlling the distribution and abundance of some Neotropical taxa remain unclear. For example, *Riethia* is an important component of some lakes in Patagonia, making up 70% of the chironomid assemblage in habitats between 10 and 12°C (Massaferro et al. 2014). In the present study, however, *Reithia/Pseudochironomus* is less common and occurs in much colder temperatures from 4 to 16°C. Similarly, *Paratanytarsus* is one of the most dominant taxa in the Andean dataset from 4 to 14°C, but is not found in any significant numbers in the modern Patagonian dataset, yet is recorded in Late Glacial/Holocene fossil records from the region (Massaferro et al. 2014).

Within our tropical dataset, several taxa show clear climatic and environmental preferences that suggest Neotropical chironomids could be useful environmental indicators from a palaeoecological perspective. For example, *Cladopelma lateralis*‐type, *Cladotanytarsus*, and *Goeldichironomus* were only found in low‐elevation warm lakes (*c*. 20–26°C) and do not extend their ranges into colder habitats (Fig. 6), whereas *Tanytarsus* type II and *Polypedilum nubeculosum‐*type are restricted to high‐elevation cold lakes (<8°C). The occurrence of taxa restricted to warm or cold environments suggests the feasibility of the development of a quantitative model for inferring temperatures from fossil chironomid records in the region.

Despite clear faunal differences between sites at different elevations and temperatures some taxa appear to tolerate a wide temperature range. Taxa such as *Chironomus anthracinus‐*type and *Limnophyes* are found in lakes across all four biomes over the entire gradient (4505 m, MAT < 2–26°C) (Fig. 6). In Patagonia, *Limnophyes* has been recorded in lakes from 3 to 17°C (Massaferro and Larocque 2013). *Chironomus anthracinus* type has often been noted to be widely distributed and will tolerate lowland eutrophic waters (Pérez et al. 2013) and high‐elevation oligotrophic lakes (Dieffenbacher‐Krall et al. 2007). Massaferro et al. (2014) found specimens of *Chironomus* in a wide range of Patagonian lakes from 8 to 17°C with peak abundances at *c*. 12–14°C. At present, it is unclear whether the wide distribution and broad ecological tolerances of these genera reflect their eurytopic ecology or because they include several cryptic species with narrower ecological tolerances.

Thirty‐nine species of *Labrundinia* are known from South America and initial diversification probably occurred in the Neotropics (Silva et al. 2015). Our samples may include several species, but our data clearly suggests the genus is indicative of warm conditions as *Labrundinia* was only found from *c*. 16 to 22°C. Similarly in Patagonia, Massaferro et al. (2014) noted *Labrundinia* in lakes warmer than 12°C with peak abundances between *c*. 14 and 15°C. Across Central America, the genus is most abundant in sites with MATs between 22 and 24°C (Wu et al. 2014).

At subfamily level, trends associated with elevation and temperature are also apparent. Chironomini are a major component of lowland assemblages, whereas Orthocladiinae dominate the assemblage of high‐elevation lakes (Fig. 7). Chironomini species are often large and require plenty of food and warm temperatures in order to complete their development (Mackey 1977). Orthocladiinae larvae, however, are often small and can complete their development with less food and relatively quickly even in cold conditions (Mackey 1977). It is clear from both modern surveys and fossil records that Orthocladiinae are often most abundant in cold habitats, whereas many Chironominae thrive in warmer temperatures (Eggermont and Heiri 2011). The elevational transition between the two subfamilies may reflect the influence of temperature and its direct effect on development rates. The altitude of this transition could provide an important threshold from which to monitor future changes in the chironomid communities of Andean lakes. Warming temperatures may cause the altitude of this transition line to rise.

In temperate regions, many chironomid species are univoltine and development rates are strongly controlled by the temperature of the warmest month (Armitage et al. 1995). Studies assessing chironomid variation and distribution across the Northern Hemisphere have predominantly found mean July air temperature to be the most significant variable explaining variation in chironomid larval assemblages (Brooks and Birks 2000; Heiri et al. 2003; Nazarova et al. 2010; Self et al. 2011; Upiter et al. 2014). However, across the tropics, the seasonal variation in temperature is small, and as a result, most species are multivoltine (Walker and Mathews 1987). Eggermont et al. (2010) noted MAT was significant in determining chironomid variation in the tropics of Africa. Wu et al. (2014) also noted MAT as the most important variable in describing the variation in Costa Rican chironomid assemblages. In extra‐tropical Patagonia, Massaferro et al. (2014) noted the combined temperatures of the three warmest months as most important. Our data show that MAT is marginally better in explaining the species assemblage variation than MJT, the warmest season (Table 3).

### Precipitation

Of the sites studied above 1000 m a.s.l. in the lake transect, those in the Ecuadorian Andes receive on average 2300 mm/year of rainfall, while further south in southern Peru and Northern Bolivia the average is 710 mm/year. North of 2°S, Andean regions experience two rainy seasons between autumn and spring in connection with the meridional displacement of the Intertropical Convergence Zone (ITCZ) over the eastern Pacific (Mitchell and Wallace 1992). This semiannual rainfall cycle fades south along the Andes into Northern Peru. During the austral summer (December–January–February) in Andean regions, monthly rainfall is about 50–150 mm. Northern areas receive slightly more rainfall than further south. However, throughout the austral winter (June–July–August), rainfall remains high in the equatorial Andes while south of northern Peru conditions are much drier (<50 mm/month) (Garreaud 2009). The regional differences in rainfall, and their subsequent effects on catchment and in‐lake process, are reflected in the chironomid distributions. Latitude, and to a lesser extent MAP and pH, is associated with axis 2 of the CCA (Fig. 9). When used as a single constraining variable, precipitation explains 10.3% of chironomid assemblage variation (*λ*_1_/*λ*_2_ = 0.9, variance explained = 10.3%). Taxa such as *Micropsectra* and *Phaenopsectra* are strongly associated with high precipitation and high latitudes, while *Paratanytarsus* is more closely associated with high pH and low precipitation. Precipitation, or the influence of precipitation on in‐lake variables such as pH, depth, and conductivity, is a strong secondary driver of chironomid assemblages in the tropical Andes. For chironomids inhabiting lakes in the extremely wet areas of the tropical montane forest (*c*. 1500–3000 m a.s.l.), we have identified precipitation as the most important environmental variable.

## Conclusions

The rate of future warming in the lower troposphere is predicted to be greatest at high elevations (Bradley et al. 2004). Over the Andes MATs have increased by 0.1°C/decade and future climate scenarios suggest that by the end of the 21st century, temperatures may have risen by as much as 4.5–5°C (Vuille et al. 2008; Urrutia and Vuille 2009). While the inner tropical Andes have received slightly more rainfall since the latter half of the 20th century, the outer tropical Andes have steadily dried (Vuille et al. 2008). Precipitation is predicted to increase during the rainy season and decrease in the dry season, amplifying the seasonal hydrological cycle in the tropical Andes (Vuille et al. 2008; Urrutia and Vuille 2009).

This study has demonstrated that MAT and MAP are the variables that explain most of the variance in the chironomid assemblages. In response to predicted climate warming, warm‐tolerant taxa will likely experience range expansion to higher elevations, while cold stenothermic taxa will experience range contraction and increased competition. Rising temperatures will be particularly detrimental to taxa of Orthocladiinae that are in low abundances in warm habitats. Orthocladiinae will likely become more restricted to high‐elevation lakes. The current transition zone between Orthocladiinae and Chironomini at *c*. 3000 m a.s.l. provides a useful baseline from which to monitor changes in chironomid community composition.

The effect of increasing precipitation on the hydrology of an individual catchment is difficult to quantify, but rising lake levels and increased runoff, causing greater sediment influx and altering water chemistry (TOC, DO), affect chironomid habitats by substrate changes, and changing lake water conductivity. As precipitation patterns change, chironomid species that show a preference for regions of high precipitation, such as *Micropsectra* and *Phaenopsectra*, will likely change their distributions. As the outer Neotropics continue to dry, these chironomids will likely become more restricted to the inner Neotropics.

The ways in which changes in temperature and precipitation alter food webs or are filtered through a system are complex, often nonlinear and difficult to predict (Petchey et al. 1999). Nevertheless, we have demonstrated the factors most affecting the chironomids of the tropical Andes are those that have the greatest probability of changing in the future. Changes to such a key component of the aquatic system will have cascading consequences for secondary consumers dependent on chironomids as valuable sources of food. In addition to the climate pressures, the lacustrine environment available for the movement of chironomid species is limited on the eastern flank of the Andes as mid‐elevation lentic habitat is sparse (Bush et al. 2011). Rapid climate changes, combined with limited habitat space may restrict the ability for chironomid populations to colonize new areas. Observations of assemblage changes could prove vital for monitoring the affects of future change in these biodiversity hotspots (Bellard et al. 2014).

Monitoring climate‐driven change within lacustrine ecosystems is of vital importance for the future conservation of these habitats. Studies such as this demonstrate the sensitivity of the tropical Andean chironomid fauna to climatic factors. Chironomids are a valuable bioindicator of the impact of climate change on freshwater ecosystems. A larger and more representative dataset, especially from mid‐altitude localities, is needed to increase understanding of chironomid species distributions and ecology. Nevertheless, our data provide some preliminary observations that can be used to monitor and predict the impact of future environmental change on freshwater ecosystems in the tropical Andes. Furthermore, this work contributes some basic autecological information urgently needed for the interpretation of chironomid palaeoecological records in the region.

## Conflict of Interest

None declared.
